# Shelf-Life Enhancement Applying Pulsed Electric Field and High-Pressure Treatments Prior to Osmotic Dehydration of Fresh-Cut Potatoes

**DOI:** 10.3390/foods13010171

**Published:** 2024-01-03

**Authors:** Maria Katsouli, Efimia Dermesonlouoglou, George Dimopoulos, Eleftheria Karafantalou, Maria Giannakourou, Petros Taoukis

**Affiliations:** Laboratory of Food Chemistry and Technology, School of Chemical Engineering, National Technical University of Athens (NTUA), 9, Iroon Polytechniou Str, 15772 Zografou, Greece; mkatsouli@chemeng.ntua.gr (M.K.); gdimop@chemeng.ntua.gr (G.D.); eleftheriaak@hotmail.com (E.K.); mgian@chemeng.ntua.gr (M.G.); taoukis@chemeng.ntua.gr (P.T.)

**Keywords:** *Solanum tuberosum*, osmotic dehydration, pulsed electric fields, high pressure, browning, fresh-cut quality, shelf life

## Abstract

From a quality standpoint, it is desirable to preserve the characteristics of fresh-cut potatoes at their peak. However, due to the mechanical tissue damage during the cutting process, potatoes are susceptible to enzymatic browning. This study pertains to the selection of the appropriate osmotic dehydration (OD), high pressure (HP), and pulsed electric fields (PEF) processing conditions leading to effective quality retention of potato cuts. PEF (0.5 kV/cm, 200 pulses) or HP (400 MPa, 1 min) treatments prior to OD (35 °C, 120 min) were found to promote the retention of the overall quality (texture and color) of the samples. The incorporation of anti-browning agents (ascorbic acid and papain) into the osmotic solution improved the color retention, especially when combined with PEF or HP due to increased solid uptake (during OD) as indicated by DEI index (2.30, 1.93, and 2.10 for OD treated 120 min, non-pre-treated, HP pre-treated, and PEF pre-treated samples, respectively). PEF and HP combined with OD and anti-browning agent enrichment are sought to improve the quality and microbial stability of fresh-cut potatoes during refrigerator storage. Untreated fresh-cut potatoes were characterized by color degradation from the 2nd day of storage at 4 °C, and presented microbial growth (total viable counts: 6 log (CFU)/g) at day 6, whereas pre-treated potato samples retained their color and microbiologically stability after 6 days of cold storage (total viable counts, <4 log(CFU)/g).

## 1. Introduction

Osmotic dehydration (OD) is attained by immersing foodstuffs in a hypertonic solution, and the main process parameters are temperature, time, solid/liquid ratio, and osmotic solution properties (water activity, formulation) [1,2]. Depending on the food product, the appropriate processing parameters should be selected based on an effective dehydration of reasonably short duration and retention of the foodstuff’s quality. As extensively discussed in the recent literature, process parameters, including temperature, process duration, and osmotic solution composition, play a crucial role in the OD effect [3,4]. In [5], the impact of the OD solutes is particularly studied, showing the beneficial effect of natural concentrates, such as concentrated juices, on final products attributes. In the same context, Kaur et al. [6] reported the benefits of employing unconventional natural sweeteners, instead of the conventional sucrose. OD as a pre-treatment step is used to increase the quality and shelf life of fresh-cut high-moisture-content fruits and vegetables [1,3,7]. From a quality standpoint, it is preferable to preserve at their peak the characteristics of value-added fresh-cut fruits and vegetables, such as potatoes from Naxos Island (Cyclades, Greece), which have protected geographical indication certifications proving their unique characteristics. All the above justifies the need for optimizing the processes that can maintain the quality and extend the shelf-life of this variety to be exported from the island and distributed to the market.

Potatoes are particularly popular among customers due to their high mineral, dietary fiber, and other nutrient content, as well as their convenient food preparation [8,9,10]. However, due to the mechanical tissue damage during the fresh-cutting process, they become prone to accelerated enzymatic browning [11]. The browning process affects the nutrient content of the potatoes and reduces shelf life, thus largely restricting the development of the fresh-cut potato processing industry [12,13]. The browning of fresh-cut fruits and vegetables is a result of the enzymatic oxidation of phenolic compounds to quinones, catalyzed by the enzyme PolyPhenol Oxidase (PPO; EC 1.14.18.1), and their subsequent condensation to colored pigments [14,15]. The effect of OD on color retention preventing the enzymatic browning of fresh-cut fruits and vegetables has been reported [1,3,16]. In the literature on osmotic dehydration of fresh-cut fruits and vegetables, no reported study on the efficacy of anti-browning agents on physico-chemical properties and the browning phenomenon of osmo-dehydrated fruits and vegetables has been reported. OD using anti-oxidants, acidifiers, and chelating agents could prove to be a minimal pre-treatment to improve the nutritional and sensory quality of fresh-cut products [17,18,19,20,21,22,23,24]. Studies on OD of potato so far have focused on the mass transfer kinetics [14], as well as on pre-treatment prior to frying, aiming to limit oil uptake [25,26,27].

Due to its stiff tissue structure, osmotic dehydration of potato is significantly hindered by slow mass transport. Novel nonthermal processing methods during or before OD (such as pulsed electric fields and high pressure) are proposed to enhance mass transfer [28]. Pulsed electric fields (PEF) have been recognized as an important pre-treatment to the traditional dehydration processes [29,30]. As far as osmotic dehydration is concerned, Kaur et al. [6] report the main advantages of performing a PEF step prior to OD, mainly focusing on obtaining improved drying rates. In some earlier research, the beneficial application of PEF was proven on apples, leading to increased mass transfer with minimum product quality deterioration [31,32]. PEF treatment was also implemented before OD of mangoes to modulate solid uptake [33]. Its effect on plant cells is based on electroporation, whereby an external electric field causes the formation of pores on the cell membrane and cell wall. Pore formation renders cells permeable, expediting the transport of intracellular material (mainly water) to the extracellular matrix [34]. This flux of water significantly accelerates dehydration but also causes loss of turgor pressure within the cells, causing tissue softening. Thus, textural degradation of plant tissues, which is a common side effect of PEF pre-treatment, occurred [35]. Therefore, the selection of appropriate PEF treatment conditions with the aim of increasing water transfer rates needs to strike a balance between effective water loss and minimal textural deterioration. The degree of plant cell breakdown caused by PEF treatment is commonly determined based on physicochemical characteristics that are affected by the treatment [34]. Plant tissue firmness is strongly correlated with the degree of electroporation and is commonly applied for the indirect assessment of the percentage of cells that have undergone electroporation. PEF application in the pre-treatment of fried potatoes has been mainly studied [36,37,38]. Researchers have been focused on applying PEF to raw potatoes to increase permeability of potato cell membranes, reduce the oil content/acrylamide content of fried potatoes, and improve texture characteristics of fried potatoes [36,38]. Apart from as a pre-treatment to OD, PEF has been extensively studied as a pre-treatment to other dehydration methods such as air drying and freeze drying of plant tissues. In the case of air drying, electroporation increases moisture transfer from the plant matrix, leading to shorter drying times or lower drying temperatures [39,40]. The mechanism of action of PEF as a pre-treatment to air drying is analogous to that of osmotic dehydration, in the sense that transfer rates of water removal are enhanced [41]. The mechanism of action of PEF as a pre-treatment to freeze drying is also attributed to the more pronounced transfer of water into the gas phase and to changes in temperature distribution within the product [42]. Enhanced drying rates, improved rehydration characteristics, and shape retention have been reported as the most significant benefits [43].

Much like PEF treatment, the exposure of plant tissues to high hydrostatic pressure (HP) (100–600 MPa) has been shown to increase plant cell permeability while controlling the activity of endogenous enzymes. The selection of adequate HP process parameters is based on a balance between effective dehydration and enzyme inactivation/quality status (color/texture) of the final food product. HP can result in better retention of fresh food characteristics (flavor, taste, and color) while achieving inactivation of target spoilage factors such as microbes and enzymes [44]. As an industrial technology, HP has also attracted extensive attention from the fruit and vegetables processing and juice industries for potential inhibition effect on browning enzymes [44,45,46]. In relevant studies, PPO was partially inactivated after HP with residual activity in the range of 5–90% [47]. During HP, the secondary and tertiary enzyme structure rupture at high pressures of >600 Mpa was linked to the phenomenon of PPO activation or inactivation [48]. Thus, HP efficiency regarding the activity of enzyme depends on various factor such as type of food, origin of enzyme, treatment duration, initial pH, and presence of certain additives in foods [48]. Regarding HP as a pre-treatment prior to OD, the effect is strongly dependent on the process conditions applied, namely pressure and time. In [49], strawberries subjected to a 400 Mpa pressure for 10 min retained well both nutritional and anti-oxidant characteristics, and similar positive results regarding quality retention were also observed in candied wu mei fruit in [50]. As a pre-treatment to OD, HP has been reported to increase mass transfer rates due to pressure-induced cell breakage [51,52]. Its effect as a pre-treatment to other drying processes such as air drying has also been attributed to the increase in cell permeabilization [53,54].

The present study aims to determine and model the effect of pulsed electric fields (PEF) and high pressure (HP) pre-treatment conditions on osmotic dehydration (OD) parameters (mass transfer) and product quality parameters (product water activity, texture and color, enzyme activity) of fresh-cut “Naxos” potatoes. The quality loss and microbial growth during cold storage of treated potatoes were evaluated. Emphasis was given to the effect on the browning of fresh-cut potatoes. For this purpose, the use of anti-browning agents in the osmotic solutions (in combination with pre-treatments) was considered.

## 2. Materials and Methods

### 2.1. Raw Material

Potatoes from Naxos Island (Spunta variety) were manually peeled (1−2 mm) and cut (10–15 g of dimensions 4 × 1 × 1 mm approximately). The initial moisture content of potatoes was 0.848 ± 0.150 g water/g initial sample weight.

### 2.2. Pulsed Electric Fields (PEF) Treatment of Potatoes

Prior to the application of pulsed electric fields (PEF) pre-treatment, a screening of alternative treatment conditions was implemented to establish the relationship between PEF treatment and quality deterioration. PEF treatments were performed on fresh-cut potatoes submerged in tap water (electrical conductivity 800 µS/cm) at an electric field strength of 0.5–2.0 kV/cm and number of pulses ranging from 0 (untreated) to 1000 (15 µs pulse width), leading to a specific energy input up to 12 kJ/kg. Treatments were performed using the ELCRACK-5 kW device (DIL, Quackenbrück, Germany) using a stainless-steel treatment chamber; the instrumentation and method is described in Andreou et al. [55]. After each treatment, color and texture (firmness) were determined (Section 2.6).

The degree of tissue damage was estimated from the electrical conductivity disintegration index (*Z_p_)*. *Z_p_* is calculated by measuring the electrical conductivity of the untreated sample and PEF treated samples at low (1 kHz) and high (1 MHz) frequencies [56,57] (Equation (1)).

(1) $${Ζ}_{p}=1-\frac{K_{b}}{K_{b}^{\prime }}\cdot \frac{K_{b}^{\prime }-K_{i}^{\prime }}{K_{b}-K_{i}}$$

where *K_ι_*, *K*′*_ι_* are the electrical conductivities of untreated and treated material, respectively, at a low-frequency, and *K_b_*, *K*′*_b_* are the electrical conductivities of untreated and treated material, respectively, at a high-frequency. The *Z_p_* index assumes values between 0 and 1 for untreated (intact) tissue and completely damaged tissue, respectively. Electrical conductivity of the untreated and treated samples was measured in a treatment chamber using the instrumentation and method described in Andreou et al. [55].

### 2.3. High-Pressure (HP) Treatment of Potatoes

High-pressure (HP) treatments were conducted at 25 °C with a pilot-scale HP device (Food Pressure Unit FPU 1.01, Resato International BV, Assen, The Netherlands) capable of reaching pressures up to 1000 Mpa and temperatures up to 90 °C. Polyethylene glycol served as the pressure transfer medium. The device was fitted with a 1.5 L pressure vessel suitable for the treatment of larger samples. Temperature was controlled using a heating/cooling mantle with water circulating from a bath to maintain stability. The adiabatic heating rate during pressurization was monitored through a CR1000 thermocouple data logger system, which recorded a 3 °C temperature increase per 100 Mpa. The temperature was quickly stabilized via the cooling system at rates of about 5 °C/min for the large vessel and 20 °C/min for the smaller vessels. Pressurization was achieved at a rate of 13 Mpa/s, and depressurization occurred almost instantly in 1–3 s. Samples were processed at 200, 400, and 600 Mpa (HP samples) for a pressurization and depressurization cycle (c. 20 s). After the treatments, enzyme activity (PPO), firmness, and color were measured based on the analytical protocols described below in Section 2.6. The suitable HP treatment conditions were chosen such that an adequate tissue alteration would occur allowing for an acceleration in dehydration rates without causing a complete textural collapse.

### 2.4. Osmotic Dehydration (OD) of Untreated and PEF/HP Pre-Treated Potatoes

Untreated and treated samples (PEF, HP pre-treatment) were osmotically dehydrated (OD) using a solution of (wt) 40% glycerol, 10% sodium chloride, and 1% ascorbic acid (AA) or 0.03% papain (P) at a liquid to food ratio of 5:1 at 35 °C for 0–180 min [58]. Glycerol was selected as the main ingredient in osmotic solutions due to its ability to act as a humectant and plasticizer enabling the preservation of the food’s moist appearance and mouthfeel. Sodium chloride enhances mass transfer [58]. Ascorbic acid is probably the most widely used anti-browning agent, as it slightly lowers pH and has reducing properties. In particular, AA reduces the o-benzoquinones back to o-diphenols, also having a direct effect on polyphenol oxidase (PPO) [59,60]. Enzymatic treatments with proteases that attack PPO have been proposed as an alternate treatment for enzymatic browning prevention. PPO suppression by proteases was thought to be due to proteolysis or binding at specific locations required for activation. Another proposed mechanism of action was the presence of sulfhydryl groups (such as cysteine) in the proteases. In this scenario, PPO inhibition could be accomplished using papain (P) [61]. The use of ascorbic acid and papain as anti-browning agents in the osmotic solution was based on results of preliminary experiments where ascorbic acid led to complete inactivation of PPO and papain to partial inhibition of PPO (by 70%) (45 °C, 60 min, anti-browning effect on PPO activity of potato extract).

Fresh-cut potatoes were pre-weighed and placed in cylindrical glass containers. The containers were filled with slightly preheated (to reach 35 °C) osmotic medium to yield a liquid to food sample ratio of 5:1. Samples were placed in a thermo-stated water bath under agitation (240 rpm) and were withdrawn at 0, 30, 60, 120, and 180 min for determination of the OD product quality parameters and mass transfer. OD samples were then rinsed with water to remove the excess solution and wiped carefully with absorbent paper. Water loss (WL), solid gain (SG), water activity (a_w_), sensory properties, and quality indices such as color and texture (firmness) evolution during OD were determined (OD time, up to 180 min).

### 2.5. Quality Monitoring of Nontreated and PEF/HP Treated Potatoes during Cold Storage

The untreated and PEF/HP treated OD potato cuts were packed into polyethylene (PE) plastic bags (250 × 175 × 0.04 mm; ten slices of potato cuts from each repetition) and stored at 4 °C (up to 6 days) in controlled temperature cabinets (Sanyo MIR 153, Sanyo Electric, Osaka, Japan). On storage day 0, 1, 2, 3, and 6, three bags of slices per processing with one bag per repetition were randomly selected for quality study. Color (browning)/texture (firmness) and microbial stability (total viable counts (TVC), yeasts/molds) of all samples were measured.

### 2.6. Analytical Protocols

#### 2.6.1. Determination and Mathematical Modelling of Mass Transfer of Osmotic Dehydration

During osmotic dehydration, water loss (WL) and solid gain (SG) of each sample were determined gravimetrically after drying at 105 °C for 24 h (WTB BINDER 7200, Type C53, Tuttlingen, Germany). Water loss and solid gain were calculated from the following equations (Equations (2) and (3)), respectively:

(2) $$WL=\frac{m_{0}-m_{0}\cdot DW_{wb}-\left(m_{wet}-m_{dry}\right)}{m_{0}\cdot DW_{wb}}$$

(3) $$SG=\frac{m_{dry}-DW_{wb}\cdot m_{0}}{DW_{wb}\cdot m_{0}}$$

where *m*_0_ is the initial wet weight of the sample before immersion in the osmotic solution, *DW_wb_* is the dry weight of the untreated sample in g water/g sample wet base, *m_wet_* is the wet weight of the treated sample in g, and *m_dry_* is the dry weight of the treated sample in g. The dehydration efficiency index (*DEI*), defined as the ratio of water loss *WL* to solid gain *SG*, was calculated.

#### 2.6.2. Determination of Physicochemical Parameters

Water activity (a_w_) was measured using a portable water activity meter (Aqua lab 4TEV, Decagon Devices, Pullman, WA, USA). Samples were equilibrated at room temperature, prior to the measurement. pH of the final samples was determined using a pH meter (338, Amel Instruments, Milano, Italy). For the analysis, 10 g of homogenized potato sample was diluted in 90 mL of sterilized Ringer solution [62].

#### 2.6.3. Determination of Potato Firmness

Potato cuts were compressed to a deformation of 20% by a knife fitted on a Texture analyzer (TA-XT2i, Stable Micro Systems, Godalming, UK) (Test speed of 1.00 mm/s; Distance 4.00 mm). The firmness was recorded as the maximum compression force (F_max_, N) [13]. At least 5 replicates were performed per measurement.

#### 2.6.4. Determination of Potato Color

An Xrite-i1 portable digital colorimeter (Gretag-Macbeth, Grand Rapids, MI, USA) was used to measure the objective color on the surface of the potatoes, and it was expressed in the CIE-Lab scale. At least five replicates were taken in order to measure color, at three equidistant equatorial points on the potatoes. Browning was expressed using the Browning Index (BI), given by the following equation (Equation (4)) [63,64]:

(4) $$BI=\frac{100\left(\frac{\left(a+1.75*L\right)}{\left(5.645*L+a-0.3012*b\right)}-0.31\right)}{0.17}$$

where *L*, *a*, and *b* are the measured CIELab color parameters.

#### 2.6.5. Determination of PPO Activity

Polyphenol oxidase (PPO) extraction was performed according to Mukherjee et al. [59].

#### 2.6.6. Microbial Growth

For microbiological enumeration, a representative sample (10 g) was transferred to a sterile stomacher bag with 90 mL sterilized Ringer solution (Merck, Darmstadt, Germany) and was homogenized for 60 s with a Stomacher (Bag Mixer, Interscience, Saint Nom la Bretêche, France). Samples (0.1 mL) of 10-fold serial dilutions of homogenates were spread on the surface of Rose Bengal Chloramhenicol (RBC, Merck, Darmstadt, Germany) for enumeration of yeasts and molds after incubation at 25 °C for 120 h. Total aerobic viable count (TVC) was enumerated on plate count agar (PCA, Merck, Darmstadt, Germany) after incubation at 25 °C for 72 h. Two replicates of at least three appropriate dilutions were enumerated. The microbial growth was then described using the Baranyi growth model. For curve fitting, the program DMFit was used (available at http://www.combase.cc/index.php/en/ (accessed on 24 July 2023)). The kinetic parameters, microbial growth rate (k in d^−1^), and lag phase (λ in d) were estimated.

### 2.7. Statistical Analysis

The means and standard deviations of three experimental duplicates were used to calculate the results. Factorial analysis of variance (factorial ANOVA) was performed to estimate the main interaction effects of the investigated factors. Duncan’s multiple range test was employed as a post hoc analysis for the separation of means with significant differences (*p* < 0.05). Statistica 7.0 (StatSoft, Inc., Tulsa, OK, USA) software was used for all statistical analyses.

## 3. Results and Discussion

### 3.1. PEF and OD Treatment of Potatoes

#### 3.1.1. PEF Effect on Potatoes

**Figure 1:**
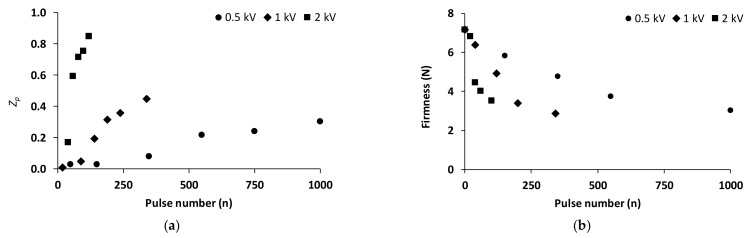
(**a**) Cell rupture index *Z_p_* of fresh-cut potatoes during PEF treatment and (**b**) firmness (Force, N) of fresh-cut potatoes, after PEF treatment (field intensity: 0.5, 1, 2 kV/cm; number of pulses: up to 1000).

The decrease in cell rupture index accurately represents the degree of electroporation generated by pulsed electric fields in plant tissues (from 0 to 1). Before PEF application as a pre-treatment to osmotic dehydration, various PEF conditions (0.5–2.0 kV/cm field strength, 0–1000 pulses of 15 µs width and 20 Hz frequency) were used in order to establish the limits within which the treatment remains effective, while over-processing was avoided. Figure 1a shows that an increase in field strength from 0.5 to 2.0 kV leads to a significant *Z_p_* value increase. At field intensity of 0.5 kV/cm, the cell rupture index assumes the value of 0.3 (approximately) for 1000 pulses. On the other hand, at field intensity of 2.0 kV/cm, the cell rupture index approaches the value of 0.9 (approximately) for less than 100 pulses. PEF pre-treatment causes turgor loss of plant tissues. Pre-treatment conditions must be carefully selected to avoid excessive softening. According to firmness measurements, PEF application significantly reduced the firmness of fresh-cut potato (Figure 1b). For <200 pulses, firmness significantly decreased as processing intensity increased and did not differ significantly with process intensity increase for >500 pulses. This indicates that a maximal level of electroporation was reached, which cannot be exceeded by increasing the pulse delivery. Additionally, it is concluded that the application of the highest intensities, of 1 and 2 kV/cm, significantly reduced the firmness of the samples, even at low pulse values. The results demonstrated that the higher electric field intensity and the higher number of pulses increased the cell rupture index but significantly reduced the tissue firmness since its cell structure was ruptured. At the same time, low intensity leads to lower energy expenditure, as the specific energy is proportional to the number of pulses and the square of the intensity of the electric field [65]. Thus, the lowest electric field strength value, equal to 0.5 kV/cm and 200 pulses, was selected for further study and analysis.

#### 3.1.2. PEF Pre-Treatment Effect on Potatoes OD

**Figure 2:**
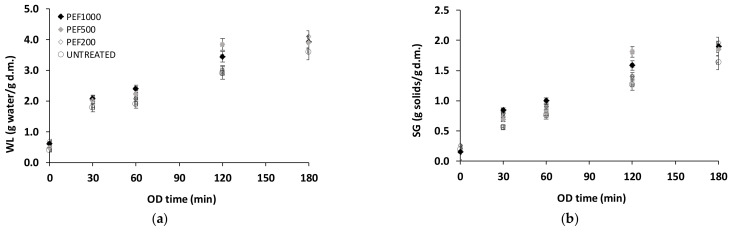
Mass transfer: (**a**) water loss (WL) and (**b**) solid gain (SG) during osmotic dehydration (OD) of untreated and PEF pre-treated (different pulse values) potatoes. Samples: Untreated, PEF200, PEF500, PEF1000 (Values are mean ± standard error).

PEF pre-treatment affects mass transfer (water loss and solid gain) and product water activity reduction during OD. In Figure 2a,b, it can be seen that at the beginning of the OD process, water loss and solid gain increased rapidly, approaching stable values after 120 min, for both non and PEF pre-treated potatoes. In the present study, PEF pre-treatment (0.5 kV; 200, 500, 1000 p; Samples: PEF200, PEF500, PEF1000) significantly affected the mass transfer during OD of potatoes (up to 180 min of OD). For longer OD times (>120 min), the application of PEF (200, 500, 1000 p) seemed to result in a more pronounced effect on mass transfer (especially solids uptake) (Figure 2b). Calculated *DEI* values ranged from 2.10 to 2.15 for OD time 120 min and from 2.06 to 2.11 for OD time 180 min of PEF pre-treated samples compared to values of 2.30 and 2.25 for OD times 120 and 180 min of untreated samples indicated that *SG* was enhanced by the electroporation achieved (PEF). The dry weight of PEF pre-treated osmotically dehydrated samples ranged from 0.529 to 0.551 g/g initial weight (compared to 0.152 g/g initial weight of the untreated sample).

PEF pre-treatment induced changes in a_w_ evolution with water activity values being less than 0.87 after 180 min of OD (Table 1). No statistically significant differences were observed between different pulse values (200, 500, 1000, Samples: PEF200, PEF500, PEF1000) (*p* > 0.05; apart from OD time 180 min where statistically significant differences were calculated between untreated and 200, 500 and 1000). It is important to note that PEF may slightly decrease the sample water activity. Although overall water activity values were lower at more intense processing conditions, the temporal evolution (rate of a_w_ decrease) was not affected by PEF treatments. This observation indicates that electroporation causes an instantaneous release of free water immediately after processing. A swift immersion in the osmotic solution suffices to remove this released free water and decrease water activity. Specifically, the water activity of potatoes, just treated with PEF, is 0.970, while the respective value for the untreated ones is 0.985. However, as confirmed by the results, after osmotic dehydration, equally low water activity values are achieved, which do not allow for microbial growth to occur. Values <0.91 are commonly adopted as the minimum water activity values where bacterial proliferation is significantly reduced. Compared to other dehydration processes, Lebovka et al. [39] found that air drying of PEF pre-treated potato tissue exhibited significantly shorter drying times due to the enhanced moisture diffusion coefficients caused by electroporation. The beneficial effects of electroporation were also reported by Liu et al. [66], who reported a decrease in oil uptake during frying of potato discs.

#### 3.1.3. PEF Pre-Treatment Effect on Potatoes Quality during OD

**Figure 3:**
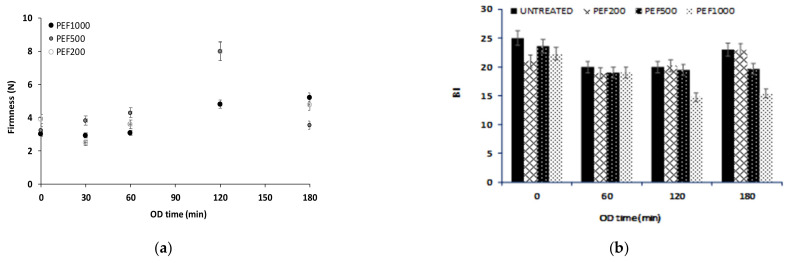
(**a**) Firmness (force, N) and (**b**) browning index (BI) values of PEF pre-treated OD potatoes during OD (0, 60, 120, 180 min). Samples: Untreated, PEF200, PEF500, PEF1000 (Values are mean ± standard deviation/error).

PEF pre-treatment affects the product quality (texture-firmness and color-browning) during OD. The firmness of potato samples after PEF is an important parameter, as it affects the sensory characteristics of the final product. In Figure 3a, the variation of the firmness of the samples in relation to the osmotic dehydration time and pulsed electric fields pulse number is presented. The softening of the potato was observed as a result of PEF and OD application (decrease in firmness value from approximately 8.5 N of the fresh-cut potato samples to 5.5 N after PEF200 and 3 N after PEF200/500/1000 and OD). However, during OD, the firmness values were increased. It was noted that 120 min of OD led to increased firmness values, approaching the firmness value of fresh-cut potato (PEF200/500 + OD). For OD time >120 min, the firmness values were significantly reduced.

Browning affects consumer acceptability of fruit and vegetable products [66]. The color degradation of treated potatoes as a function of osmotic dehydration time and pulsed electric fields pulse number is expressed by the browning index (BI) (Figure 3b). BI values of PEF pre-treated OD samples were lower than the respective values of OD samples (untreated). Given the promising results of PEF pre-treatment, the addition of anti-browning agents (ascorbic acid or papain) in the osmotic solution will be studied to further reduce the BI.

### 3.2. HP and OD Treatment of Potatoes

#### 3.2.1. HP Effect on Enzymatic Activity of Potatoes

Regarding the results of enzyme activity, it was observed that 38% of PPO inactivation was achieved after 5 min of HP treatment at 600 MPa. It seems that further increase in the processing time from 5 to 20 min does not affect the enzyme activity. Similar results (approximately 40% PPO inactivation) were reported by Van Buggenhout et al. for potato enzyme extract as well as potato cuts (100–500 MPa, 20 °C, 13 min) [67]. Tsikrika et al. reported that potato PPO inactivation was estimated to be 18–40% for pressure 600 MPa and processing times 5–20 min [68,69].

#### 3.2.2. HP Pre-Treatment Effect on Potatoes OD

**Figure 4:**
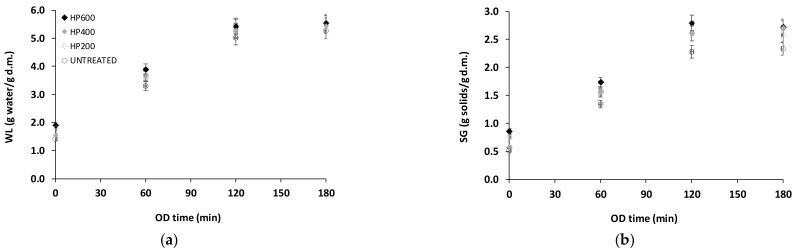
Mass transfer: (**a**) Water loss (WL: g water/g d.m.) and (**b**) solid gain (SG: g solids/g d.m.) during osmotic dehydration (OD) of HP pre-treated potatoes. Samples: Untreated, HP200, HP400, HP600 (values are mean ± standard error).

Figure 4 presents the dehydration curves in terms of water loss (WL) and solid gain (SG) for untreated and HP treated samples. HP pre-treatment significantly affected both water loss and solid gain of osmotically dehydrated potatoes. A 36% and 53% (approximately) increase in WL and SG, respectively, was calculated for 120 min of OD of HP treated samples. WL and SG remained stable after 120 min of OD treatment for both untreated and HP treated samples. It seems that HP increased the cell permeability and facilitated to some degree the diffusion during osmotic dehydration of potato by increasing both moisture and solid diffusion (Figure 4a,b) [70,71]. In the present study, HP treatment enhanced SG more than WL. Calculated *DEI* values ranged from 1.93 to 2.03 for OD times 120 and 180 min of HP treated samples compared to values of 2.30 and 2.25 for OD times 120 and 180 min of untreated samples, indicating that *SG* was enhanced by the high pressures applied (no statistically significant differences were detected between HP pre-treated samples at 200 (HP200), 400 (HP400), and 600 (HP600) Mpa regarding DEI index; *p* > 0.05). The dry weight of HP pre-treated osmotically dehydrated samples ranged from 0.446 to 0.473 g/g initial weight (compared to 0.152 g/g initial weight of the untreated sample). Sopanangkul et al. reported that the diffusion coefficient of sucrose in potatoes significantly increases with pressure up to 600 MPa at 20 and 40 °C [70]. Dourado et al. confirmed that application of appropriate levels of pressure (100–400 MPa) can be used to accelerate mass transfer during ingredient infusion into potato tissue and to reduce processing times. According to Dourado et al., applying proper levels of pressure (100–400 MPa) can be used to speed mass transfer during ingredient infusion into potato tissue and shorten processing times [8]. They concluded that higher pressures above 400 MPa induce starch gelatinization and hinder diffusion. In the present study, no statistically significant differences were observed between HP treated samples under different processing conditions (200, 400, 600 MPa; Samples HP200, HP400, HP600). The effectiveness of HP as a pre-treatment to air drying has also been demonstrated at pressures as low as 200 MPa [55]. On the contrary, Kingsly et al. [71] reported that air drying of pineapple slices was enhanced only for treatment pressures exceeding 500 MPa. Similar observations were made by Santos et al. [72] for air drying of cashew slices. The exact effects that HP treatment has on subsequent processing are therefore highly dependent both on the type of plant tissue treated and on the dehydration method used.

High pressure and osmotic dehydration resulted in high-quality potatoes of low a_w_ (from 0.97 initial value to 0.803 (HP400)–0.838 (HP200)) (Table 2). The lowest a_w_ value after HP pre-treatment was achieved at 400 MPa (0.836 and 0.803 for 120 and 180 min of osmotic dehydration, respectively). Statistically significant differences were observed between different pressures (200, 400, 600 MPa) (*p* < 0.05; apart from 0 min and 60 min OD time).

#### 3.2.3. HP Pre-Treatment Effect on Potatoes Cuts Quality during OD

**Figure 5:**
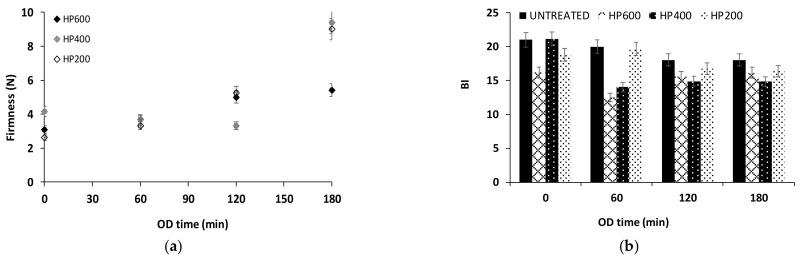
(**a**) Firmness (force, N) and (**b**) browning index (BI) of HP pre-treated potatoes. Samples: UNTREATED, HP200, HP400. HP600 (Values are mean ± standard deviation/error).

In Figure 5a, the change in firmness (F_max_, N) as a function of osmotic dehydration time and pressure is presented. It is observed that the firmness value was significantly reduced immediately after HP treatment (time 0), indicating that high-pressured potatoes were significantly softer than the fresh-cut potatoes (decrease in firmness value from approximately 7.8 N of the fresh-cut potato samples to 4 N after HP400 and OD and 3 N after HP200, HP400, HP600, and OD). The firmness increased with OD time (after 120 min of OD) as a result of solid uptake (Figure 5a). HP treated (200, 400 MPa) and OD potatoes (after 180 min of OD) were harder compared to fresh-cut potatoes. Similar behavior was noticed for the PEF pre-treated OD potatoes. Kingsly et al. [71] also reported a textural deterioration of HP pre-treated pineapple slices during air drying, which suggests that this effect may not be unique to the case of OD.

In Figure 5b, the color change (expressed as browning, BI index) is presented as a function of osmotic dehydration parameters (time and pressure). HP pre-treated samples at 400 and 600 MPa showed less browning compared to HP pre-treated samples at 200 MPa. Given the promising results of HP pre-treatment, the addition of ascorbic acid or papain as anti-browning agents in the osmotic medium will be studied to further reduce the BI.

### 3.3. HP or PEF and OD Treated Potatoes

The quality stability, in terms of browning, as well as the microbial stability of nontreated, HP osmo-dehydrated, and PEF osmo-dehydrated (with addition of the anti-browning agents: ascorbic acid or papain) potatoes was monitored as a function of storage days.

#### 3.3.1. Quality Stability (Browning) of HP or PEF and OD Potatoes during Storage

**Figure 6:**
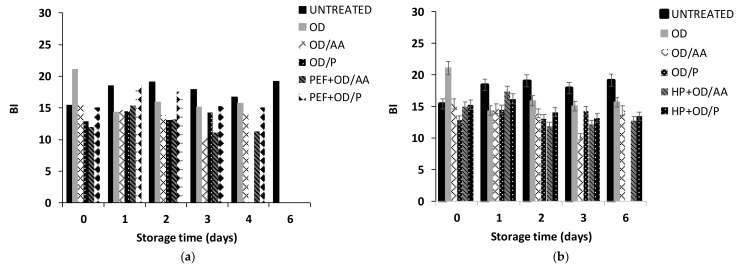
Browning index (BI) of (**a**) UNTREATED, OD (OD, OD/AA, OD/P) and PEF pre-treated OD (PEF+OD/AA, PEF+OD/P) and (**b**) UNTREATED, OD (OD, OD/AA, OD/P), and HP pre-treated OD (HP+OD/AA, HP+OD/P) potatoes during storage at 4 °C (values are mean ± standard error).

BI of untreated (control), osmo-dehydrated (OD, OD/AA, OD/P), PEF osmo-dehydrated (PEF+OD, PEF+OD/AA, PEF+OD/P), and HP osmo-dehydrated (HP+OD/AA, HP+OD/P) potatoes were monitored during cold storage (4 °C) (Figure 6a,b). BI of OD samples was calculated to be 70% lower than the BI of untreated samples and 28.5% lower than the BI of OD/AA samples (non-PEF pre-treated). The use of the anti-browning agents, ascorbic acid (AA) and papain (P), in the osmotic solution (both non-PEF and PEF pre-treated) improved the color retention for PEF pre-treated samples (OD/AA, OD/P and PEF+OD/AA, PEF+OD/P compared to OD). PEF+OD/P samples presented an increase in the browning rate compared to PEF+ODAA that can be attributed to the partial inhibition of PPO by papain (by 70%) compared to the complete inactivation of PPO using ascorbic acid.

Regarding the effect of HP pre-treatment on browning of OD potatoes, HP+ODAA/P potato samples presented higher BI values than the respective OD potato samples (ODAA/P). The increased BI of HP osmo-dehydrated samples could be attributed to an increase in enzymatic browning caused by a release of cell contents containing intracellular enzymes and their substrates (normally separated by compartmentalization in fruits) into an extracellular mass allowing better enzyme–substrate interaction. Significantly higher BI values were calculated for OD potato samples compared to OD/AA and OD/P potato samples. The use of the anti-browning agents, ascorbic acid, and papain in the osmotic solution improved the color retention (OD/AA, OD/P compared to OD) (Figure 6b). OD/AA and OD/P samples (followed by HP+OD/AA-OD/P samples) presented improved overall visual quality (based on browning index), reduced browning/discoloration, and surface dehydration compared to nontreated samples.

**Figure 7:**
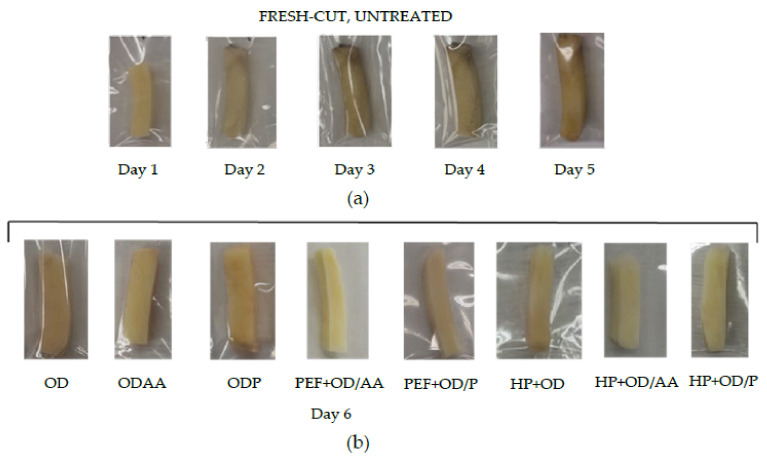
(**a**) Untreated fresh-cut potatoes stored at 4 °C for 6 days, and (**b**) OD (OD, OD/AA, OD/P), PEF pre-treated OD (PEF+OD/AA, PEF+OD/P) and HP pre-treated OD (HP+OD, HP+OD/AA, HP+OD/P) potatoes during storage (at 4 °C).

Concluding, HP and PEF+OD/AA presented the lowest BI value (lighter color). More specifically, HP and PEF reduced the BI index by 12.5% and 27.9%, respectively, compared to the samples that have only been osmo-dehydrated. In the case of papain-treated (OD/P) samples, HP enhanced the mass transfer phenomena leading to further retention of potato product color. However, the difference between BI values of all treated samples is small. The potato variety (Spunta) studied for the fresh market in southern Europe, the Middle East, and North Africa, is one of the most susceptible varieties to browning [73]. Thus, for such a sensitive tissue as that of this particular variety, the color differences obtained by the application of the two nonthermal technologies combined with osmotic dehydration could be considered significant. Figure 7a shows the color change of the nontreated potatoes as a function of storage time at 4 °C and Figure 7b for the osmo-dehydrated potatoes after 6 days of storage at 4 °C (OD, OD/AA, OD/P, HP+OD/AA, HP+OD/P, PEF+OD/AA, PEF+OD/P). The color of the ODAA+PED, OD/P+PEF, and OD/AA potato samples was lighter up to the 6th day of storage, compared to both the untreated and OD/P samples. The raw potatoes on the 2nd day of their storage showed some black spot over a larger area. However, it can be seen that the BI value of the untreated sample was stable after the 2nd day of storage, with a difference of 0.5% compared to the 6th day of storage. Even though color measurements were made at three representative points of potato tissue, a failure of the BI index to reflect the color difference is detected. For this reason, visual inspection is useful for more accurate monitoring of processing. Cantos et al., in a study on the enzymatic browning of potato, evaluated the color development both through the BI index and by organoleptic control due to the specificity of the tissue, and indeed differences were noted between the results [73].

#### 3.3.2. Microbial Growth of HP or PEF and OD Potatoes during Cold Storage

Fresh-cut fruits and vegetables are particularly susceptible to microbial growth due to the preliminary processes used (e.g., peeling, cutting, and slicing), in addition to their physicochemical, sensory, and nutritional qualities [74].

**Figure 8:**
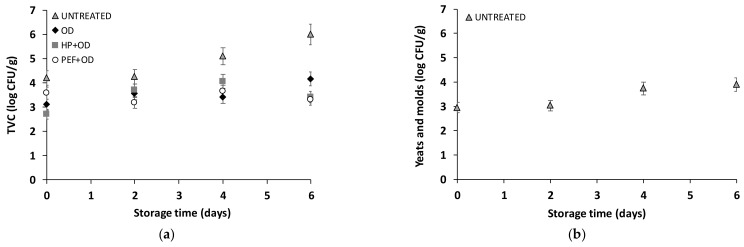
Microbial growth (**a**) total viable counts (TVC) and (**b**) yeasts and molds, log(cfu/g) of (**a**) Untreated fresh-cut potatoes during cold storage (at 4 °C) (Day: 1 to 5, from left to right) and OD (OD, OD/AA, OD/P), PEF pre-treated OD (PEF+OD/AA, PEF+OD/P), and HP pre-treated OD (HP+OD, HP+OD/AA, HP+OD/P) potatoes at Day 6 (Values are mean ± standard deviation).

Figure 8 shows the change in total microbial load of nontreated and PEF/HP pre-treated OD potatoes during cold storage. Microbial growth (total viable counts TVC) was observed for nontreated potato samples equal to 4 log(CFU)/g after two days of storage and 6 log(CFU)/g at the end of storage. The growth rate was calculated to be k = 0.569 d^−1^ (Baranyi model; DMfit programe (IFR, Institute of Food Research, Reading, UK) [75]. The total viable counts for OD treated (non-pre-treated and PEF/HP pre-treated) were <4 log(CFU)/g for 6 days of cold storage. Yeasts and molds in OD treated (non-pre-treated and PEF/HP pre-treated) samples were at undetectable levels (<10 CFU/g). In the case of the untreated sample, yeast growth was observed after 2 days of storage, with a value of 3.74 log(CFU)/g at the end of storage.

The antimicrobial effect of the osmotic dehydration process may be attributed to the hindrance of possible microorganism penetration through the cellular spaces because the intercellular spaces are filled with concentrated osmotic solution [76]. Glycerol, which was selected as the main water activity lowering agent in the osmotic medium, also showed advantages as a microbiological protectant. The water activity values of developed osmo-dehydrated ensures this microbial stability.

## 4. Conclusions

The scope of this study was to explore the application of PEF, HP, and OD for effective treatment of potatoes with the aim of retaining or improving quality of the fresh-cut counterpart. Based on the results obtained, PEF pre-treatment prior to OD (35 °C, 20 min) with an electric field strength of 0.5 kV/cm and the application of 200 pulses was found to lead to increased mass transfer while preserving the overall quality of the samples during subsequent storage. The application of HP as a pre-treatment for OD results in the production of products of acceptable quality. A treatment at 400 MPa (for 1 min) was established to be suitable for this purpose, since it accelerates dehydration while preserving the majority of most measured quality attributes. Ascorbic acid and papain as the selected anti-browning agents inhibited polyphenol oxidase, increasing shelf life and consumer acceptability of processed raw potato products by preserving the color. The incorporation of anti-browning agents into the osmotic solution (OD) improved the color retention, especially when combined with PEF or HP due to increased solid uptake during OD. Non-thermal processes, such as PEF and HP combined with osmotic dehydration and anti-browning agent, are sought to improve quality stability of fresh-cut potatoes and further increase the shelf-life of fresh-cut potatoes. PEF/HP+OD can minimize the detrimental effects on fruits/vegetable quality to meet the consumer demand of nutritious high quality fresh-cut foods with an extended shelf-life.

## Figures and Tables

**Table 1:** Water activity (a_w_) reduction during osmotic dehydration (OD) (OD time: 0, 60, 120, 180 min) after pulsed electric fields (PEF) treatment. Samples: Untreated, PEF200, PEF500, PEF1000.

OD Time (min)	Sample Water Activity (a_w_) *			
	PEF Pre-Treated 200 Pulses (PEF200)	PEF Pre-Treated 500 Pulses (PEF500)	PEF Pre-Treated 1000 Pulses (PEF1000)	Untreated
0	0.9705 ± 0.0088 Aa	0.9697 ± 0.0045 Aa	0.9726 ± 0.0056 Aa	0.9684 ± 0.0078 Aa
60	0.9486 ± 0.0079 Bb	0.9500 ± 0.0089 Bb	0.9533 ± 0036 Bb	0.9387 ± 0.0069 Bb
120	0.9189 ± 0.0025 Cc	0.9187 ± 0.0036 Cc	0.9157 ± 0.0077 Cc	0.8947 ± 0.0072 Cc
180	0.8320 ± 0.0063 Dd	0.8659 ± 0.0080 De	0.8562 ± 0.0082 De	0.8345 ± 0.0063 Dd

* Values are mean ± standard error. Values assigned with the same capital and small letter in the same column and row (A, B, C, D and a, b, c, d, e) are not statistically significant, regarding the OD time and the PEF pulse number on the presented a_w_ values, respectively (*p* < 0.05). Initial a_w_ value: 0.9861 ± 0.0052.

**Table 2:** Water activity (a_w_) reduction in potatoes during osmotic dehydration (OD) (OD time: 0, 60, 120, 180 min) after high-pressure (HP) treatment. Samples: Untreated, HP200, HP400, HP600.

OD Time (min)	Sample Water Activity (a_w_) *			
	HP Pre-Treated 600 Mpa (HP600)	HP Pre-Treated 400 Mpa (HP400)	HP Pre-Treated 200 Mpa (HP200)	Untreated
0	0.9663 ± 0.0120 Aa	0.969 ± 0.0067 Aa	0.9733 ± 0.0094 Aa	0.9733 ± 0.0105 Da
60	0.9060 ± 0.0109 Bb	0.9166 ± 0.0075 Bb	0.9273 ± 0.0068 Bbc	0.9387 ± 0.0121 Bc
120	0.8434 ± 0.0101 Ca	0.8364 ± 0.0121 Ca	0.8836 ± 0.0120 Cb	0.8947 ± 0.0098 Cb
180	0.8052 ± 0.0096 Da	0.8033 ± 0.0090 Da	0.8382 ± 0.0089 Db	0.8345 ± 0.0078 Db

* Values are mean ± standard error. Values assigned with the same capital and small letter in the same column and row (A, B, C, D and a, b, c, d) are not statistically significant regarding the OD time and the PEF pulse number on the presented a_w_ values, respectively (*p* < 0.05). Initial a_w_ value: 0.9861 ± 0.0052.

## Data Availability

The data presented in this study are available on request from the corresponding author. The data are not publicly available due to project grant confidentiality restrictions.
